# The Neuroprotective Role of Polydatin: Neuropharmacological Mechanisms, Molecular Targets, Therapeutic Potentials, and Clinical Perspective

**DOI:** 10.3390/molecules26195985

**Published:** 2021-10-02

**Authors:** Sajad Fakhri, Mohammad Mehdi Gravandi, Sadaf Abdian, Esra Küpeli Akkol, Mohammad Hosein Farzaei, Eduardo Sobarzo-Sánchez

**Affiliations:** 1 Pharmaceutical Sciences Research Center, Health Institute, Kermanshah University of Medical Sciences, Kermanshah 6734667149, Iran; sajad.fakhri@kums.ac.ir; 2 Student Research Committee, Kermanshah University of Medical Sciences, Kermanshah 6714415153, Iran; mehdigravandi@yahoo.com (M.M.G.); abdian.ph@gmail.com (S.A.); 3 Department of Pharmacognosy, Faculty of Pharmacy, Gazi University, 06330 Ankara, Turkey; esrak@gazi.edu.tr; 4 Department of Organic Chemistry, Faculty of Pharmacy, University of Santiago de Compostela, 15782 Santiago de Compostela, Spain; 5 Instituto de Investigación y Postgrado, Facultad de Ciencias de la Salud, Universidad Central de Chile, Santiago 8330507, Chile

**Keywords:** polydatin, neurodegeneration, neuroprotection, therapeutic targets, pharmacology, novel delivery system

## Abstract

Neurodegenerative diseases (NDDs) are one of the leading causes of death and disability in humans. From a mechanistic perspective, the complexity of pathophysiological mechanisms contributes to NDDs. Therefore, there is an urgency to provide novel multi-target agents towards the simultaneous modulation of dysregulated pathways against NDDs. Besides, their lack of effectiveness and associated side effects have contributed to the lack of conventional therapies as suitable therapeutic agents. Prevailing reports have introduced plant secondary metabolites as promising multi-target agents in combating NDDs. Polydatin is a natural phenolic compound, employing potential mechanisms in fighting NDDs. It is considered an auspicious phytochemical in modulating neuroinflammatory/apoptotic/autophagy/oxidative stress signaling mediators such as nuclear factor-κB (NF-κB), NF-E2–related factor 2 (Nrf2)/antioxidant response elements (ARE), matrix metalloproteinase (MMPs), interleukins (ILs), phosphoinositide 3-kinases (PI3K)/protein kinase B (Akt), and the extracellular regulated kinase (ERK)/mitogen-activated protein kinase (MAPK). Accordingly, polydatin potentially counteracts Alzheimer’s disease, cognition/memory dysfunction, Parkinson’s disease, brain/spinal cord injuries, ischemic stroke, and miscellaneous neuronal dysfunctionalities. The present study provides all of the neuroprotective mechanisms of polydatin in various NDDs. Additionally, the novel delivery systems of polydatin are provided regarding increasing its safety, solubility, bioavailability, and efficacy, as well as developing a long-lasting therapeutic concentration of polydatin in the central nervous system, possessing fewer side effects.

## 1. Introduction

Neurodegenerative diseases (NDDs) are amongst the most common factors of disability and death in humans, which refer to the gradual, symmetrical, and specific decreases in sensory, motor, and mental nerve activity resulting in the death of neurons [1,2]. Nerve death accounts for various signs of neurological dysregulations, both chronic and acute, consisting of Parkinson’s disease (PD), Alzheimer’s disease (AD), central nervous system (Brain/Spinal Cord) injuries, and stroke [3]. Additionally, autism, neuropathic pain, aging, and depression are other NDDs that result from nerve cell death [4,5]. From a mechanistic point of view, various factors cause neurological problems, such as oxidative stress [6], inflammation [7], and apoptosis [5,8]. The aforementioned pathological pathways play a harmful role in neuronal cell death mechanisms. The microglia activity, inflammatory cytokines, reactive oxygen species (ROS), and related mitochondrial disruption of oxidative pathways have shown negative results on the process of nerve regeneration that eventually leads to cell death [9,10].

Despite advances in clinical healthcare, neuroprotective agents are still clinically challenged in nerve destruction and NDDs. Thus, there is an emerging need to develop new multi-target therapies that further help to attenuate dysregulated signaling pathways in NDDs [11,12,13]. Several natural compounds isolated from edible and medicinal plants that exhibit anti-inflammatory properties have been investigated for potential application as pharmaceutical candidates [14]. Natural products are rich sources of polyphenolic compounds, consisting of stilbenoids, which are a big group of resveratrol substances such as monomers, dimers, and oligomers. Stilbenoids are naturally occurring compounds in a variety of plant families, such as Vitaceae, Gnetaceae, Cyperaceae, and Rocarpaceae. Consequently, the wine grape, *Vitis vinifera* L., is considered the primary nutritional source of these compounds [15].

Polydatin is a stilbenoid that passively penetrates cells. It also launches into the cells through an active mechanism by a glucose carrier. The glucose moiety of polydatin causes a higher resistance rate to enzymatic oxidation than resveratrol and has much better water solubility [16,17]. Polydatin has been shown to suppress oxidative stress, inflammation, and apoptosis as major pathways for nerve cell regeneration. The biological activity of polydatin and certain derivatives entails preventing or interfering with several neurodegenerative mechanisms [18].

In a previous study, the protective mechanisms of polydatin were evidenced in cerebral ischemia [19]. Recently, dementia-related disorders are also targeted by polydatin [20]. Besides, the general pharmacology and pharmacokinetic properties of polydatin were developed by Du et al. [21]. As of yet, no review article has discussed the entire set of neuroprotective mechanisms of polydatin. This review focuses on the pharmacological targets, molecular mechanisms, therapeutic potentials, and clinical perspectives of polydatin in NDDs. The pharmacological mechanisms of action of polydatin in the treatment or prevention of NDDs are provided.

## 2. Polydatin: Chemical Structure, Sources, and Pharmacokinetic Properties

**Figure 1:**
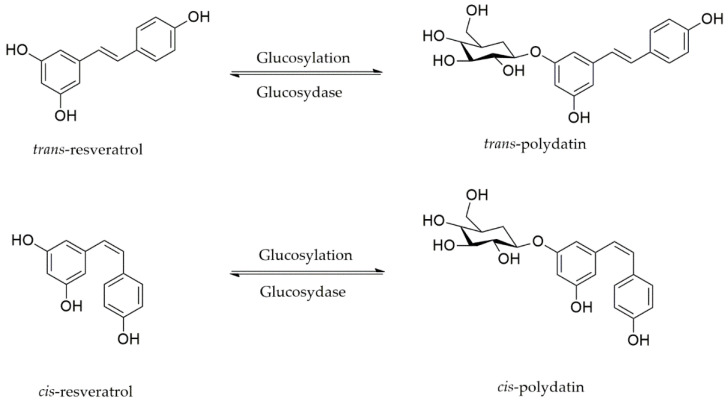
Polydatin, a glycosylated form of resveratrol.

Several studies concerning the chemical characterization of stilbenoids have been motivated by their numerous promising biological functions, especially those of polydatin. Polydatin (3,4′,5-trihydroxystilbene-3-β-d-glucoside) is a natural resveratrol glucoside known as resveratrol-3-β-mono-d-glucoside, an active product from the *Polygonum cuspidatum* Sieb. et Zucc roots (Figure 1). However, it is also found in grapes, red wines, hop cones, peanuts, cocoa/chocolate products, and several other meals [21].

Two isomeric types (*cis* and *trans*) of polydatin are found in nature. *Cis*-polydatin is often detected in lower levels. Moreover, they are less biologically active than the *trans* forms [22]. The most common sources of polydatin are grape juice and red/white wines. *Cis*-polydatin is the predominant isoform in carbonated wines and rosé, while the *trans* isomer is abundant in berries, peanuts, grapes, and pistachios [23]. The major sources of polydatin isomers are the rhizomes and roots of *Fallopia japonica* (Houtt.) Ronse Decraene (Polygonaceae), which have long been used in traditional Chinese and Japanese Medicine as an anticancer, diuretic, analgesic, anti-pyretic, and expectorant agent in the management of atherosclerosis [24]. However, this product is present in various other genera such as *Rumex, Picea, Rosa, Quercus,* and *Malus*. Polydatin has received similar consideration to resveratrol because glucoside concentrations are usually higher than aglycone ones in red wine and other grape products. The exact ratio of glycosylated forms to aglycones in wine relies on various aspects such as the fermentation method and ecological conditions in the vineyards [25].

Pharmacokinetic studies are often required for the effective and safe clinical use of drugs. The absorption, distribution, and metabolism of polydatin are connected to its bioactivity. Polydatin might have higher bioavailability and a better antioxidant function compared to resveratrol. In addition, intestinal absorption of polydatin is higher than resveratrol made by glucose groups [26]. Polydatin enters the cell through an active glucose carrier mechanism and passive diffusion, while resveratrol just passively penetrates cell membranes [27]. The active transport of polydatin mainly passes through a sodium-dependent glucose transporter 1 (SGLT1), chiefly present in the intestines and stomach [16]. Since the cell content of polydatin is not very low, it indicates an active transfer of polydatin by SGLT1 [21,27].

Polydatin employs two possible pathways to be deglycosylated from *trans*-resveratrol. The primary pathway is cleavage by cytosolic-β-glucosidase following the SGLT1 mediated by passing through the brush-border membrane. The second mechanism, which happens on the luminal side of the epithelium, is deglycosylation by the membrane-bound enzyme lactase-phlorizin hydrolase. This mechanism is followed by passive diffusion of the released aglycone and additional glucuronoconjugation [17]. Although resveratrol is more accumulated and leaves more residue in cells than polydatin, the half-life of polydatin is approximately four hours with a higher level of resveratrol Cmax at the same dose [27]. However, more analytical methods need to be investigated for the determination of *trans*-stilbene glycoside during pharmacokinetics studies [28].

Accordingly, polydatin as a glycosylated resveratrol could be a potential therapeutic agent with fewer pharmacokinetic limitations in comparison to resveratrol.

## 3. Polydatin against NDDs

Polydatin has demonstrated several biological/pharmacological effects, such as anti-inflammatory [29], anti-apoptotic [30], and antioxidant [31], against NDDs [32]. To combat oxidative stress, polydatin increased antioxidant capacity through associated antioxidant mediators, nuclear factor erythroid 2-related factor 2 (Nrf2) and sirtuin 1 (Sirt1), and antioxidant response elements (AREs) [18]. Polydatin suppresses oxidative stress through phosphoinositide 3-kinases (PI3K)/protein kinase B (Akt)-interconnected mediators [33]. It also blocks oxidative stress and reduces microglial apoptosis through the Nrf2/heme oxygenase (HO-1) pathway [34]. From the inflammatory point of view, by suppressing nuclear factor kappa B (NF-κB), polydatin can stop intercellular adhesion molecule-1 (ICAM-1) protein/mRNA production. Polydatin has also been shown to reduce pro-inflammatory cytokines (IL-1β, TNF-α, and IL-6) by down-regulating toll-like receptor-2 (TLR-2) and the NF-κB p65 pathway [35]. As mitochondria are the major source of ROS in cells, when the intracellular mitochondria are damaged, electron transfer is abnormal, and ROS production is increased, which ultimately accelerates the onset of apoptosis [36]. Several studies have shown the beneficial influence of polydatin on mitochondria from a new perspective. Polydatin has been considered to suppress mitochondria-related cytochrome c release, moreover suppressing caspase-9 and caspase-3 [37]. Polydatin has been thought to decrease ROS release and improve mitochondrial activity by modulating the Sirt3/superoxide dismutase 2 (SOD2) pathway. SOD2 is a mitochondrial antioxidant enzyme whose activity is mediated by Sirt3 [38].

Overall, by modulating several mediators in inflammatory/apoptotic/autophagy/oxidative stress pathways, polydatin could be a hopeful candidate in combating NDDs.

### 3.1. Polydatin against AD, and Cognition/Memory Dysfunction

As the most common form of NDDs, AD is characterized by a gradual decline in memory and mental impairment in all aspects of a person′s ability to perform daily activities, with unknown causes [39]. Studies have shown that the accumulation of old extracellular plaques, mainly consisting of the amyloid beta peptide (Aβ) and intracellular fiber nodules composed of hyperphosphorylated proteins, plays an essential role in the neuropathology of AD [40,41,42]. Besides, several inflammatory, apoptotic, and oxidative pathways are behind the pathogenesis of AD. Due to numerous pathophysiological mechanisms for AD, effective treatment has not yet been developed. Natural products have shown beneficial therapeutic effects on AD [43]. Amongst natural entities, oral administration of polydatin could dramatically reduce the production of malondialdehyde (MDA) and increase the activity of the antioxidants SOD and catalase (CAT) to protect learning and memory impairments in vivo. In addition, it lessened the damage caused by an oxygen-glucose deficiency in cultured neurons [44]. Tong et al. investigated the protective effect of polydatin in cancer patients undergoing chemotherapy, most of whom had cognitive impairments due to the use of chemotherapy drugs. In their study, polydatin, at a daily dose of 50 mg/kg, reduced doxorubicin-induced cognitive impairment and restored the hippocampal structure of the hippocampus. In addition, polydatin reduced doxorubicin-induced stress by regulating Nrf2, activating the NF-κB pathway, and reducing apoptosis [45,46]. In another study, polydatin was reported to defend against learning and memory failure in neonatal rats with hypoxic-ischemic brain injury (HIBI) caused by unilateral carotid artery ligation. In addition, polydatin decreased memory deficiency and increased the expression of the hippocampal brain-derived neurotrophic factor (BDNF) in rats with HIBI [47]. Moreover, in a study on rat’s cognitive function exposed to chronic ethanol, polydatin increased cell survival while decreasing the expression level of cyclin-dependent kinase 5 (cdk5), and reversed functional defects in ethanol-treated mice evaluated by the Morris water test [48]. In another recent study, polydatin has shown protective roles against dementia-related disorders by attenuating several dysregulated pathways, including suppressing neuroapoptosis, oxidative stress, N-methyl D-aspartate receptor subtype 2B (NR2B), senile plaques, neurofibrillary tangles, and cholinergic dysfunctions [20]. Polydatin-mediated in vitro inhibition of Aβ25–35 polymerization and associated fibrils/oligomers was also reported by Rivière et al. [49,50]. As another anti-AD mechanism of polydatin, an in vitro increase in α3 and α7 nicotinic acetylcholine receptors (nAChRs) could help combat NDDs [51]. During an in vivo study, the modulation of NR2B by polydatin in rats’ prefrontal cortex reduced learning and memory impairments [52].

Therefore, polydatin could be a helpful candidate in preventing AD and cognitive/memory impairment in various cases. Such an effect is exerted through the modulation of several dysregulated mechanisms, including neurological deficit scores, oxidative stress (e.g., Nrf2, SOD, CAT), inflammation (e.g., NF-κB), as well as Aβ, BDNF, and nAChRs.

### 3.2. Polydatin against PD

PD is an aging-associated condition and the second-most significant reason for NDDs [53]. PD is known for midbrain dopaminergic neuronal loss and the accumulation of α-synucleins called Lewy bodies. Furthermore, damages to non-dopaminergic pathways cause non-motor and motor malfunctions [54]. Owing to their poor effectiveness and adverse side effects, traditional therapies for PD are challenging to implement, and the development of novel innovative and safe agents is now needed. Oxidative stress and neuroinflammation play a significant role in PD pathogenesis [55]. Therefore, preventing the dysregulated mediators of these pathways has a considerable role in prohibiting the dissemination of PD. From a pathophysiological perspective, the degradation of substantia nigra dopaminergic neurons is caused by the hereditary sensitivity and response to harmful environmental stimuli [56]. Bai et al. reported that polydatin could play a critical role in combating PD. Besides, polydatin meaningfully decreased apoptosis and mitochondrial dysfunction during rotenone/Parkin deficiency induced in a human dopaminergic neuronal cell line, SH-SY5Y. In their study, polydatin suppressed the rotenone-induced cell death, mitochondrial membrane potential (MMP), Sirt 1, DJ1, and ROS production. Their study found that when autophagy-related gene 5 (Atg5) is biologically inhibited, the beneficial effects of polydatin are partly inhibited, implying Atg5-mediated neuroprotection [57]. Parkin knockdown-induced oxidative stress, mitochondrial malfunction autophagy deficiency, and mitochondrial fusion expansion were all alleviated by polydatin [58]. Polydatin therapy may also reverse abnormalities in mitochondrial morphology and motor malfunction in a Drosophila model of PD caused by Parkin insufficiency [57].

In the pathogenicity of PD, neuroinflammation hyperactivates microglia and results in the destruction of dopaminergic neurons. As a result, reducing microglial activity could help in the management of PD [59]. Polydatin crosses the blood–brain barrier to protect motor deterioration of substantia nigra and preserves dopaminergic neurons and motor function by suppressing pro-inflammatory mediators and microglia [60,61]. Huang et al. indicated that polydatin caused an increase in Nrf2, p-Akt, and p-glycogen synthase kinase-3β (GSK-3β) Ser9, activated microglial BV-2 cells, and suppressed NF-κB and pro-inflammatory mediators in the substantia nigra of PD rat-induced by lipopolysaccharide (LPS). Polydatin also inhibited dopaminergic neurodegeneration caused by microglial activation through modulating the Akt/GSK-3β/Nrf2/NF-κB signaling pathway [62]. It is worth noting the discrepancies on the anti/pro-inflammatory cytokines following microglia activation. It reveals the complexity of the brain microglial regulation, including the critical M1 (inflammatory microglia) and M2 (anti-inflammatory microglia). Microglia activations, especially the M1 type, have been considered a critical orchestrator in triggering inflammatory responses during NDDs. However, the production/release of inflammatory cytokines has been highlighted as a common feature associated with the microglial response, which is closely related to imbalanced protein homeostasis in NDDs [63]. So, modulating microglia activation could be a promising strategy for polydatin in combating NDDs.

The disturbance of glycolysis and the decrease in ATP production are other factors involved in the dysfunction of dopaminergic neurons and developing PD [64]. Zhang et al. showed that polydatin might improve glycolysis, glucose metabolism, ATP production, and motor dysfunction in mice with 1-methyl-4-phenyl-1,2,3,6-tetrahydropyridine (MPTP)-induced early dopaminergic neuronal degeneration. In their study, polydatin prevented the loss of dopaminergic neurons in the striatum and substantia nigra, thereby suppressing neural apoptosis (Bax and cleaved caspase-3) and improving motor function in mice [65]. Suppressing complex I of the electron transport chain and heightened oxidative stress are among the first triggers in the pathogenesis of PD [66]. In an in vitro study, reducing lipid peroxidation, inhibiting apoptosis, and activating the mitogen-activated protein kinase (MAPK) are introduced as the primary neuroprotective mechanisms of polydatin on dopaminergic neurons [67]. A study by Ahmed et al. showed that polydatin (3 mg/kg, intraperitoneally) possessed a neuroprotective effect in attenuating the degeneration of dopaminergic neurons in nigro-striatal regions of the brain. They also indicated that polydatin improved neuromotor behavior in a rat model of rotenone-induced PD. Thus, the protective effect of polydatin against striatal degeneration is presented in their report [68]. In a similar report, polydatin meaningfully prevented the rotenone-induced dysregulations of MDA, manganese SOD, glutathione, and thioredoxin in the striatum. Besides, polydatin inhibited the rotenone-induced neurodegeneration of dopaminergic neurons in the substantia nigra [61].

Polydatin, as a balancer, may thus be a treatment strategy in PD by reducing oxidative stress, as well as controlling autophagic mechanisms and mitochondrial fusion.

### 3.3. Polydatin against Central Nervous System (Brain/Spinal Cord) Injuries

Traumatic brain injury (TBI) is the leading cause of corporality, and permanent dysfunction has become a global public health problem [69]. People with severe TBI sometimes necessitate lengthy therapy. Treatments are missing due to the complexity and obscurity of the pathophysiological pathways in TBI [70]. TBI induced mitochondrial neuronal damage, as evidenced by an increase in ROS mitochondria and a reduction in MMP, causing the previous mitochondrial transition pore to open [69]. Polydatin has shown various pharmacological benefits, including antioxidation, anti-inflammation, anti-apoptosis, and brain-associated injuries [71,72].

Sprague–Dawley rats receiving 30 mg/kg polydatin intraperitoneally after TBI decreased in ROS and blocked TBI-induced MDA expression while increasing SOD levels in damaged cortices. In their study, polydatin prevented MMP collapse and the previous mitochondrial transition pore from opening TBI and reduced the endoplasmic reticulum stress response following TBI [69]. Consistently, polydatin significantly lowered endoplasmic reticulum stress-related unfolded protein activation, containing blocked p-extracellular regulated kinase (ERK) phosphorylation, declined spliced XBP-1, and cleaved activating transcription factor 6 (ATF6) production, as well as increasing the expression of glucose-regulated proteins (GRP78). Besides, polydatin regulated the p38MAPK signaling pathway and the mitochondrial apoptotic pathway (e.g., caspase-3/9) and improved neurological scores and the length of survival in TBI rats [69]. In another report, polydatin protected against SCI by suppressing oxidative stress and apoptosis passing through Nrf2/HO-1 signaling in vitro and in vivo [34]. Polydatin also increased neuronal viability and protected against oxygen-glucose deprivation/re-oxygenation-induced mitochondrial injury and apoptosis in a dose-dependent manner. Besides, polydatin modulated the activity of neuronal mitochondria, including MMP, intracellular calcium levels, the opening of the mitochondrial permeability transition pore (mPTP), ROS generation, and adenosine triphosphate levels. From a mechanistic perspective, polydatin suppressed Keap1 and upregulated Nrf2/HO-1 and NAD(P)H Quinone Dehydrogenase 1 (NQO-1) in oxygen-glucose deprivation/re-oxygenation-treated spinal cord motor neurons. Additionally, polydatin reversed the mitochondrial and neuronal damage induced by spinal cord ischemia/reperfusion in a mouse model, partially suppressed by the Nrf2 inhibitor. This represents that the neuroprotective effects of polydatin pass through the Nrf2/ARE pathway [73]. The engagement of Nrf2 on neuronal differentiation in both in vivo and in vitro studies are also provided by Zhan et al. [74]. The involvement of Nrf2/ARE in the protective effects of polydatin is also presented in other reports [75]. In this line, the inhibitory effect of polydatin on ferroptosis was shown both in vitro and in TBI mice. Those responses were applied by preventing the accumulation of free Fe^2+^, increasing MDA, and decreasing glutathione peroxidase (GPx) [76].

The most common causes of traumatic spinal cord injury (SCI) are motor/car collisions, abuse, and falls [77]. Not unexpectedly, epidemiological trials discovered that SCI mainly existed in young males and resulted in lifelong cognitive defects that significantly reduce their life quality [78]. SCI is characterized by various symptoms, including limb paralysis, a loss of feeling in the lower extremities, and uracratia or uroschesis. A growing body of research suggests the aggregation of inflammatory cytokines across the compromised spinal cord and is amongst the main risk aspects for SCI pathological symptoms [10,11]. Findings indicated that several pro-inflammatory cytokines, including the macrophage migration inhibitory factor (MIF), interleukin-1 (IL-1), IL-6, and tumor necrosis factor-α, are intensified steadily after compression-induced SCI [9]. To modulate these mechanisms, polydatin was injected into adult male Sprague–Dawley rats in a single intraperitoneal dose. In this line, polydatin significantly reduced spinal cord edema and morphological changes in vivo. It also decreased nitric oxide (NO) in spinal cord tissues of SCI rats, which was consistent with the pattern of inducible nitric oxide synthase (iNOS) production. Accordingly, LPS increased protein and mRNA levels of iNOS in BV2 cells, and polydatin reversed these changes [78]. Consequently, polydatin decreased the LPS-induced rise in NO and response to inflammatory microglia. Polydatin also significantly reduced IL-6, IL-1, and TNF-α after a single injection and inhibited the development of inflammatory cytokines in spinal cord tissues following SCI. Besides, polydatin blocked LPS-induced NF-κB activation in BV2 microglia and inhibited the activity of NLRP3 inflammasomes [78]. This stilbene attenuated TBI-induced acute lung injury by suppressing the S100B-mediated formation of neutrophil extracellular traps [79]. Polydatin also meaningfully decreased MDA while increasing SOD, GPx, CAT, and the level of total antioxidant capacity in the brain and liver. Besides, polydatin reduced inflammatory mediators of serum, such as IL-6, IL-1β, and TNF-α. It also modulated the D-galactose-induced caspase-3 and Bcl-2/Bax ratio elevation in the liver and brain [30].

Altogether, the critical role of polydatin in the modulation of Nrf2/ARE, ERK/MAPK, and interconnected apoptotic/inflammatory pathways could pave the road in the modulation of brain/SCI injuries.

### 3.4. Polydatin against Stroke: As a Coupled Complication to NDDs

Stroke is one of the most severe cerebrovascular disorders, affecting patients’ quality of life [80]. Further pieces of evidence and mechanisms of polydatin protect against cerebral ischemia. Two different shreds of evidence have been mentioned, namely the inhibition of the neurological deficit score and limiting the brain infarction volume in rats with middle cerebral artery occlusion after being treated with polydatin. Several mechanisms have been provided for these two effects of polydatin [81].

Ischemic stroke increases neuroinflammation and ROS. Shah et al. investigated the neuroprotective activity of polydatin against ischemic brain damage in a rat model of chronic middle cerebral artery occlusion (MCAO). Their results indicated that polydatin minimized infarction volume and mitigated neurobehavioral defects by limiting the activation of p38MAPK and c-Jun N-terminal kinase, thereby suppressing neuroinflammation and ROS. They also demonstrated that polydatin upregulated the endogenous antioxidants Nrf2, HO-1, and the thioredoxin pathway, and reduced inflammation and ROS in cortical tissue [82]. As previously mentioned, inflammation and oxidative stress are two major factors in cerebral ischemic pathogenesis. In this line, NF-κB activation plays a critical role in inflammation. Besides, low levels of glioma-associated oncogene Patched-1 (Ptch1), homolog1 (Gli1), and SOD1 will lead to oxidative stress. Ji et al. demonstrated that polydatin could protect the brain of rats with permanent MCAO. Such effects were exerted by modulating inflammation via lowering NF-κB and the attenuation of oxidative stress through increasing Ptch1, Gli1, SOD1 expression, as well as ameliorating blood–brain barrier permeability [83]. Besides, the neuroprotective effects of polydatin on neurological function and the Nrf2 pathway of rats with cerebral hemorrhage were identified. Their study showed that polydatin enhanced neurological function and decreased oxidative stress in rats by controlling the Nrf2/ARE pathway and downstream gene production [84]. Mitochondrial dysfunction and apoptosis are involved in the process of ischemic stroke. In the study of Gao et al., the neuroprotective effect of polydatin was evaluated. Their results demonstrated the anti-apoptotic effect of polydatin and improved mitochondrial dysfunction due to ischemic/reperfusion injury in a rat MCAO model. Increasing Bcl-2 and decreasing cytochrome c, Bax, and caspases-3/9 are centrally associated protective mechanisms [37].

Considering the role of cell adhesion molecules (CAMs) in developing ischemia/reperfusion-induced cerebrovascular diseases in a rat MCAO model, Cheng et al. found that polydatin can reduce the volume of brain infarction by decreasing the levels of CAMs in comparison to the control group, as well as the involvement of E-selectin, L-selectin, integrins, ICAM-1, and vascular cell adhesion molecule-1 (VCAM-1) [85]. Metastasis-associated lung adenocarcinoma transcript 1 (MALAT1) is a non-coding RNA that has a role in protecting the blood–brain barrier after an ischemic event. In the study of Ruan et al., it has been demonstrated that polydatin could upregulate the expression of MALAT1. Polydatin initiated a MALAT1/CREB/PGC-1α/PPARγ cascade that eventually led to protecting cerebrovascular endothelium and blood–brain barrier integrity from ischemia [81]. Moreover, Chen et al. discovered that high doses of polydatin could reduce edema, inflammation, and apoptosis after an ischemic event in the brain tissue of rat models with MCAO by regulating the expression of p53 and Notch1. The scores for the neurological function and behavioral scores were also improved in such models [86]. During an in vitro study, the protective effects of polydatin have also been shown in influencing the regulation of neuroglobin (Ngb) promotor activity and mRNA expression [87]. Polydatin might also regulate gene expression of Ngb through the attenuation of CREB, HIF-1α, p56, and early growth response protein 1 (Egr1). Besides, a polydatin-associated reduction in NO was also related to Ngb up-regulation [88,89]. From another point of view, polydatin meaningfully inhibited cerebral edema in cerebral hemorrhage rats by suppressing excitatory amino acids [90].

Beyond the stroke, polydatin has shown several other neuroprotective effects. For instance, in the study of Guan et al., polydatin potentially showed anxiolytic effects and suppressed neuroinflammation in a chronic pain mouse model by reducing pro-inflammatory cytokines, including TNF-α and IL-1β in the amygdala [91].

Different mechanisms are employed by polydatin to combat stroke and anxiety, including Nrf2/HO-1/ARE, Bax/caspases, Egr1/Ngb, CREB, and PGC-1. Additionally, antioxidant activity, an improvement in mitochondrial health, free-radical scavenging, anti-apoptotic/anti-inflammatory activities, up-regulation of BDNF/Shh/Ngb pathway, and down-regulation of CAMs are other protective mechanisms of polydatin [19,92].

**Figure 2:**
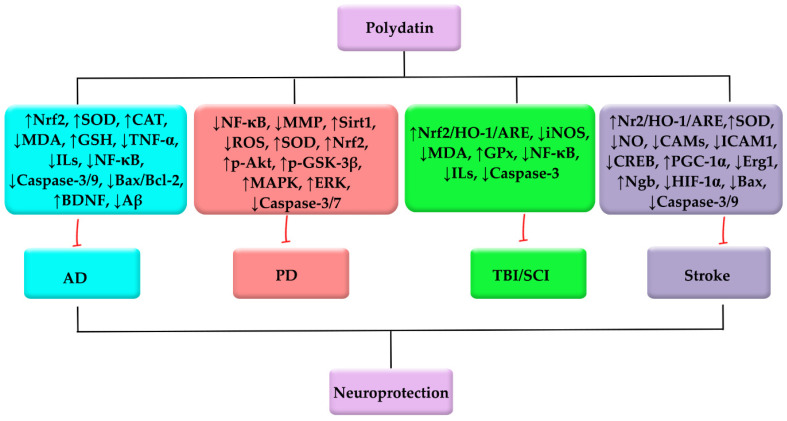
Polydatin employs several mediators to combat PD, AD, TBI/SCI, and stroke.

The entire set of neuropharmacological characteristics of polydatin against AD, PD, TBI/SCI, and stroke are presented in Table 1. Overall, by employing several mechanisms and the modulation of various dysregulated pathways, polydatin could be a promising neuroprotective phytochemical against PD, AD, TBI/SCI, and stroke (Figure 2).

## 4. Polydatin Novel Delivery Systems: Nanoformulations, and Targeted Therapy

Nanomedicine is the medicinal use of nanotechnology that employs biocompatible, low-toxicity nanomaterials and nanoparticles to control drug pharmacokinetics, administration rate, and bioavailability [96]. In addition, polydatin may guard against brain injury, kidney problems, heart failure, and improve glucose and lipid metabolism [97,98]. However, therapeutic activities of polydatin are constrained due to weak water solubility, the chemical imbalance in aqueous alkaline medium, and substantial first-pass metabolism. To address these limitations, recyclable nanostructures have sparked wide attention because of their potential in drug delivery and successful removal from the body [11]. In this way, chitosan-loaded nanoparticles administrated daily by gastric intubation for about one month improved the effect of polydatin in male Wistar albino rats [99].

In diabetes mellitus (DM), polydatin was used because of its various therapeutic mechanisms consisting of controlling free-radical production and mitochondrial activity, as well as regulation of inflammation and oxidative stress [97,98]. The anti-hyperglycemic and antioxidant effects of polydatin resulted in a substantial reduction in hemoglobin A1C in treated diabetic rats, and treatment resulted in a significant increase in hepatic glycogen levels, which may be secondary to improved insulin levels and intervention [98].

Apart from its low water solubility, the reduced effectiveness and safety risk of polydatin must be addressed before being used in clinical trials. In this way, microenvironment-sensitive nanoparticles have shown considerable promise in increasing the bioavailability of lipophilic substances [100]. The depletion of liver fibrosis in mice given a polydatin-loaded micelle (PD-MC) was verified by measuring hydroxyproline and fibrotic parameters, including collagen type 1 (Col1), tissue inhibitor of metalloproteinases 1 (TIMP-1), transforming growth factor-beta (TGF-β), and PD-MC, which not only inhibited hepatocyte apoptotic cell death but also showed anti-inflammatory properties. The anti-inflammatory activity of PD-MC was linked to its ability to suppress the ROS and TLR4/NF-B p65 signaling pathway. The mice treated with PD-MC had significantly less hepatic oxidative stress due to the lower levels of 4-Hydroxynonenal (4-HNE) [101].

Polydatin has a clear impact on the cardiac system, acting as an anticoagulant, anti-inflammatory, anti-atherosclerotic, anti-hypercholesterolemic, and anti-ischemic agent. It reduces platelet accumulation, increases microcirculation, strengthens the endothelium and nervous system, and relieves coughing and asthma, which can be found to manage shock [21]. However, the limited oral bioavailability (half-life 8–14 min) and low solubility (the highest solubility is estimated to be 30 g/mL in water at 25 °C) of polydatin has restricted its administration [21,102]. Accordingly, liposomes have shown increased solubilization and stabilization while also providing good drug concentrations for water-soluble and lipid-soluble medicines. The polydatin-loaded liposomes (10 mg/kg) system was balanced in Sprague–Dawley rats. The long-lasting characteristics of the polydatin-loaded liposomal system can improve the absorbance of polydatin in the digestive system, but there are no organ histopathologic modifications after treatment with the polydatin-loaded liposome [102].

In cancer, the traditional treatment options, such as surgery, chemotherapy, radioactivity, immunotherapy, and hormonal treatments, are inadequate for controlling cancer progression [103]. In this way, polydatin possesses various properties such as anti-proliferative, antioxidant, anti-inflammation, and immunomodulatory. For improving the anticancer effectiveness of polydatin and other novel therapies, the production of nanoparticles has received much attention [104]. So, oral administration of polydatin-loaded poly (lactic-co-glycolic acid) [PLGA] nanoparticles (polydatin-PLGA-NPs) in Syrian hamsters resulted in lower amounts of lipid peroxidative byproducts. Polydatin-PLGA-NP therapy decreased tumor histological symptoms from extreme to mild and blocked the development of squamous cell carcinoma. Besides, the administration of polydatin-PLGA-NPs led to a substantial reduction in tumor volume and occurrence. Polydatin-PLGA-NPs significantly increased enzymatic antioxidant rates such as SOD, CAT, and GPx, while decreasing the rate of cytochrome (Cyt) p450, Cyt b5, glutathione S-transferase, gammaglutamil transferase, and glutathione reductase activities, which are among the metabolizing enzymes of phases I and II. Polydatin-PLGA-NPs treatment caused apoptosis via sheared caspase-3 overexpression and the prevention of dimethyl benzyl anthracene-induced mutant p53 and cyclin-D1 production in a dose-dependent manner [105]. As another disorder, irritable bowel syndrome is currently thought to result from dysfunction in the brain-–gut axis, including both central and peripheral pathways concerned, and in particular, involving cannabinoid receptors and affecting the activity of most cells. To modulate these dysregulated mechanisms, the effect of a co-micronized form of palmitoylethanolamide/polydatin was examined in 157 patients with irritable bowel syndrome [106].

**Figure 3:**
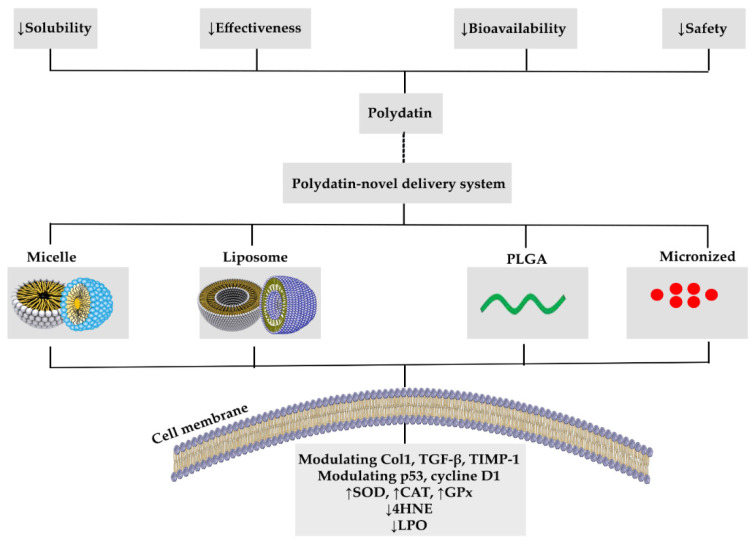
Novel delivery systems of polydatin: Reduction in the pharmacokinetic limitations.

Altogether, in addition to its high effectiveness and the more appropriate pharmacokinetic characteristics of polydatin, using novel delivery systems for this secondary metabolite could increase the associated efficacy and reduce some of the remaining limitations of phytochemicals, by increasing solubility/bioavailability and decreasing safety risks. Figure 3 shows the novel delivery systems of polydatin.

## 5. Conclusions

**Figure 4:**
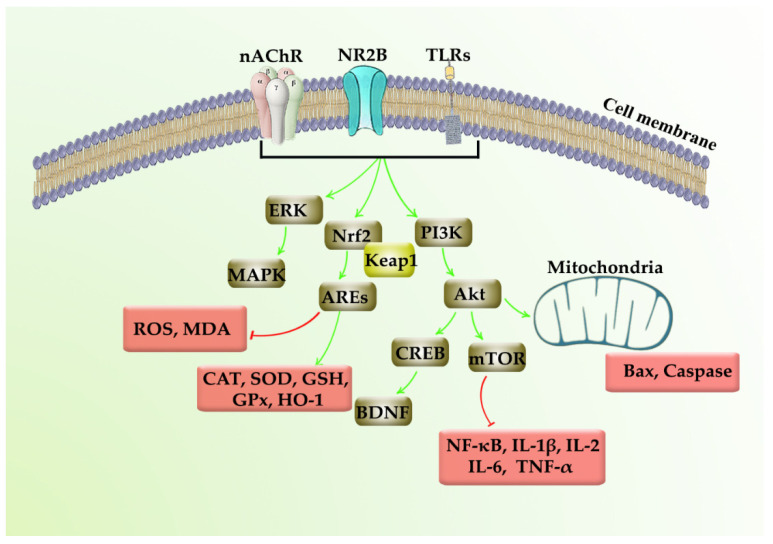
Neuroprotective mechanisms of polydatin.

Polydatin is a multi-target stilbenoid secondary metabolite extracted from herbal sources. As polydatin is a glycosylated form of resveratrol, several biological activities and health benefits are connected to the administration of polydatin, including cardioprotective, hepatoprotective, and neuroprotective factors. Prevailing studies focus on the neuroprotective potential of polydatin by employing several mechanisms, including Nrf2/Keap1/ARE, PI3K/Akt, ERK/MAPK, TLR/NF-κB/TNF-α/ILs, and Bax/Bcl-2/caspases (Figure 4). In this line, polydatin critically modulates inflammatory, apoptotic, and oxidative mediators towards combating AD, PD, stroke, CNS injuries, and miscellaneous neuroprotective responses. On the other hand, the pharmacokinetic drawbacks of polydatin, including their poor bioavailability, low solubility/selectivity, low plasma concentration, rapid metabolism, and chemical degradation, limit the associated therapeutic uses. It reveals the importance of novel drug delivery systems to reduce the restrictions in modulating tumor cell senescence. It is also worth noting that providing a novel delivery system could potentially help the polydatin to pass through the blood–brain barrier and develop a long-lasting therapeutic concentration of drugs in the CNS, while possessing fewer side effects [107,108,109].

In the present study, the pharmacological targets, molecular mechanisms, and therapeutic potentials of polydatin are highlighted through the attenuation of inflammatory/apoptotic/oxidative pathways to tackle multiple dysregulated pathways in NDDs. The need to provide novel delivery systems of polydatin, including nanoformulations, and targeted therapy is also considered. Further pre-clinical studies are needed to elucidate the precise neuroprotective mechanisms of polydatin followed by well-controlled clinical trials.

## Figures and Tables

**Table 1:** Neuropharmacological mechanisms of polydatin against different NDDs.

NDDs	Methods	Models	Neuropharmacological Mechanisms	References
**AD**	Chronic cerebral hypoperfusion	in vivo: Sprague–Dawley rats	↓MDA, ↑CAT, ↑SOD	[44]
	Doxorubicin-induced cognitive impairment	in vivo: Sprague–Dawley rats	↓Nrf2, ↑NF-κB, ↓caspase-3, ↓caspase-9	[45,46]
	HIBI	in vivo: Sprague–Dawley rats	↓Memory deficient, ↑BDNF	[47]
	Chronic ethanol exposure	in vivo: Sprague–Dawley rats	↑Cell survival, ↓cdk5, ↓functional defects	[48]
	Polymerization of Aβ	in vitro: Aβ_25–35_	↓Aβ25–35 polymerization	[49,50]
	D-galactose-induced	in vitro: DPPH in vivo: Male Kunming mice	↑Body weight, ↓MDA, ↑CAT, ↑SOD, ↑GSH, ↓IL-1β, ↓TNF-α, ↓IL-6, ↓ Bax/Bcl-2, ↓caspase-3	[30]
**PD**	Rotenone-induced	in vitro: Human neuroblastoma SH-SY5Y	↓Mitochondrial dysfunction, Atg5-mediated autophagy, modulating MMP, ↑Sirt 1, ↓ROS	[57]
	LPS-induced	in vitro: Microglial BV-2 cells in vivo: Wistar rats	↑p-GSK-3β, ↑p-Akt, ↑Ser9, ↑Nrf2, ↓NF-κB	[62]
	MPTP-induced	in vivo: Adult male BALB/c mice	↑Glycolysis, ↑ATP production, ↓motor dysfunction	[65]
	Rotenone-induced	in vivo: Sprague–Dawley rats	↑ATP, ↑SOD, ↑thioredoxin,	[61]
		in vitro: Dopaminergic SH-SY5Y cells	↑MAPK, ↓caspase-3, caspase-7, ↓LPO, ↑ERK1/2/5	[67]
**TBI/SCI**	Brain injury	in vivo: Wistar albino male rats	↓MDA, ↑antioxidant potential	[93]
	SCI	in vitro: murine microglia BV2 cells in vivo: Sprague-Dawley rats	↑Nrf2, ↑HO-1, ↓caspase-3, ↓Bax/Bcl-2 ratio	[34]
	Oxygen glucose deprivation/re-oxygenation-induced mitochondrial injury	in vivo: C57BL/6J mice in vitro: SMNs	↑Intracellular calcium levels, ↑mPTP, ↓ROS, ↓apoptosis, ↑ATP, ↓Keap1, ↑Nrf2, ↑HO-1, ↑NQO-1	[73]
	Neuronal differentiation of BMSCs	in vivo: C57BL/6 mice in vitro: Bone marrow mesenchymal stem cell (BMSC)	↑Nrf2	[74,75]
	Secondary damage of TBI	in vivo: TBI mouse model in vitro: Neuro2A cells	↓GPx, ↑MDA, ↓accumulation of free Fe^2+^	[76]
	SCI	in vivo: Sprague-Dawley rats in vitro: Murine microglia BV2 cells	↓TNF-α, ↓IL-1β, ↓NO, ↓iNOS, ↓ IL-6, ↓NF-κB	[94]
	D-galactose-induced	in vivo: Male Kunming mice in vitro: DPPH	↓TNF-α, ↓IL-1β, ↓IL-6, ↓ caspase-3, ↓Bax/Bcl-2	[30]
**Stroke**	MCAO	in vivo: Sprague–Dawley rats	↑Nrf2, ↑HO-1, ↓ROS, ↓p38, ↑Gli1, ↑Ptch1, ↑SOD1	[82,83]
	Intracerebral hemorrhage	in vivo: Wistar rat	↑Neurological function, ↑NO, ↑SOD, ↑MDA, ↑GSSG, ↑GSH, ↑Nrf2	[95]
	MCAO	in vivo: Sprague–Dawley rats	↑Bcl-2, ↓IL-1β, ↓TNF-α, ↓ IL-6, ↓Bax, ↓caspases-3/9	[37]
	Ischemia–reperfusion injury	in vivo: Sprague–Dawley rats	↓CAMs, ↓E-selectin, ↓L-selectin, ↓ICAM-1	[85]
	OGD	in vitro: Human embryonic kidney cells (HEK-293T) in vivo: Sprague–Dawley rats	↑MALAT1, ↑CREB, ↑PGC-1α	[81]
	MCAO	in vivo: Sprague–Dawley rats	↓Edema, ↓apoptosis, p53/Notch1 modulation	[86]
	OGD	in vitro: PC12 cell	↓CREB, ↓HIF-1α, ↓p56, ↓Egr1, ↑Ngb, ↓NO	[87,88,89]
	Hypoxia/ischemia and oxidative stress-induced injury	in vitro: N2a cells	↓CREB, ↑BDNF, ↑Shh, ↑Ngb, ↓apoptosis	[19,92]

AD: Alzheimer’s disease, Akt: Protein kinase B, Atg5: Autophagy Related 5, ATP: Adenosine triphosphate, Aβ: Amyloid beta, Bcl-2: B-cell lymphoma 2, BDNF: Brain-derived neurotrophic factor, BMSCs: Bone marrow mesenchymal stem cell, CAT: Catalase, Cdk5: Cyclin dependent kinase 5, DPPH: 2,2-diphenyl-1-picrylhydrazyl, Egr1: Early growth response 1, ERK: Extracellular-signal-regulated kinase, GRP78: Glucose-regulated protein, GPx: Glutathione peroxidase, GSH: Glutathione, GSK-3β: Glycogen synthase kinase-3β, GSSG: Glutathione disulfide, HEK-293T: Human embryonic kidney cells, HIBI: Hypoxic-ischemic brain injury, HIF-1α: Hypoxia-inducible factor 1-alpha, HO-1: Heme oxygenase-1, ICAM-1: Intercellular adhesion molecule-1, IL: Interleukin, iNOS: Inducible nitric oxide synthase, LPO: Lipid peroxidation, LPS: Lipopolysaccharides, MALAT1: Metastasis associated lung adenocarcinoma transcript 1, MAPK: Mitogen-activated protein kinase, MCAO: Middle cerebral artery occlusion, MDA: Malondialdehyde, MMP: Matrix metalloproteinase, MPTP: 1-methyl-4-phenyl-1,2,3,6-tetrahydropyridine, NF-κB: Nuclear factor kappa-light-chain-enhancer of activated B cells, Ngb: Neuroglobin, NO: Nitric oxide, Nrf2: Nuclear factor E2-related factor 2, OGD: Oxygen-glucose deprivation, PD: Parkinson’s disease, PTCH1: Protein patched homolog 1, ROS: Reactive oxygen species, SCI: Spinal cord injury, SMNs: spinal motor neurons, SOD: Superoxide dismutase, TBI: Traumatic brain injury, TNF-α: Tumor necrosis factor α.

## Abbreviations

AD	Alzheimer’s disease
Akt	Protein kinase B
Atg5	Autophagy Related 5
ATF6	Activating transcription factor 6 (ATF6)
ATP	Adenosine triphosphate
Aβ	Amyloid beta
Bcl-2	B-cell lymphoma 2
ARE	Antioxidant response element
BDNF	Brain-derived neurotrophic factor
BMSCs	Bone marrow mesenchymal stem cell
CAMs	Cell adhesion molecules
CAT	Catalase
Cdk5	Cyclin dependent kinase 5
Col1	Collagen type 1
Cyt	Cytochrome
DM	Diabetes mellitus
DPPH	2,2-diphenyl-1-picrylhydrazyl
Egr1	Early growth response 1
ERK	Extracellular-signal-regulated kinase
Gli1	Homolog1
GRP78	Glucose-regulated protein
GPx	Glutathione peroxidase
GSH	Glutathione
GSK-3β	Glycogen synthase kinase-3β
GSSG	Glutathione disulfide
HEK-293T	Human embryonic kidney cells
HIBI	Hypoxic-ischemic brain injury
HIF-1α	Hypoxia-inducible factor 1-alpha
HO-1	Heme oxygenase-1
ICAM-1	Intercellular adhesion molecule-1
IL	Interleukin
iNOS	Inducible nitric oxide synthase
LPO	Lipid peroxidation
LPS	Lipopolysaccharides
MALAT1	Metastasis associated lung adenocarcinoma transcript 1
MAPK	Mitogen-activated protein kinase
MCAO	Middle cerebral artery occlusion
MDA	Malondialdehyde
MIF	Macrophage migration inhibitory factor
MMP	Mitochondrial membrane potential
MPTP	1-methyl-4-phenyl-1,2,3,6-tetrahydropyridine
nAChRs	Nicotinic acetylcholine receptors
NDDs	Neurodegenerative diseases
mPTP	Mitochondrial permeability transition pore
NF-κB	Nuclear factor kappa-light-chain-enhancer of activated B cells
Ngb	Neuroglobin
NO	Nitric oxide
Nrf2	Nuclear factor E2-related factor 2
NQO-1	NAD(P)H Quinone Dehydrogenase 1
OGD	Oxygen-glucose deprivation
Ptch1	Patched-1
PD	Parkinson’s disease
PD-MC	Polydatin-loaded micelle
PI3K	Phosphoinositide 3-kinases
PLGA	Poly (lactic-co-glycolic acid)
ROS	Reactive oxygen species
SCI	Spinal cord injury
SD	Sprague–Dawley
SGLT1	Sodium-dependent glucose transporter
Sirt1	Sirtuin 1
SMNs	Spinal motor neurons
SOD	Superoxide dismutase
TBI	Traumatic brain injury
TGF-β	Transforming growth factor-beta
TIMP-1	Tissue inhibitor of metalloproteinases 1
TLR	Toll-like receptor
TNF-α	Tumor necrosis factor α
VCAM-1	Vascular cell adhesion molecule-1
4-HNE	4-Hydroxynonenal
6-OHDA	6-hydroxydopamine
